# Overview about the localization of nanoparticles in tissue and cellular context by different imaging techniques

**DOI:** 10.3762/bjnano.6.25

**Published:** 2015-01-23

**Authors:** Anja Ostrowski, Daniel Nordmeyer, Alexander Boreham, Cornelia Holzhausen, Lars Mundhenk, Christina Graf, Martina C Meinke, Annika Vogt, Sabrina Hadam, Jürgen Lademann, Eckart Rühl, Ulrike Alexiev, Achim D Gruber

**Affiliations:** 1 Institute of Veterinary Pathology, Freie Universität Berlin, Robert-von-Ostertag-Str. 15, 14163 Berlin, Germany; 2 Institute of Chemistry and Biochemistry - Physical and Theoretical Chemistry, Freie Universität Berlin, Takustr. 3, 14195 Berlin, Germany; 3 Department of Physics, Institute of Experimental Physics, Freie Universität Berlin, Arnimallee 14, 14195 Berlin, Germany; 4 Department of Dermatology, Charite - Universitätsmedizin Berlin, Charitéplatz 1, 10117 Berlin, Germany

**Keywords:** fluorescence lifetime imaging, fluorescence microscopy, histopathology, light microscopic autoradiography, structured illumination microscopy

## Abstract

The increasing interest and recent developments in nanotechnology pose previously unparalleled challenges in understanding the effects of nanoparticles on living tissues. Despite significant progress in in vitro cell and tissue culture technologies, observations on particle distribution and tissue responses in whole organisms are still indispensable. In addition to a thorough understanding of complex tissue responses which is the domain of expert pathologists, the localization of particles at their sites of interaction with living structures is essential to complete the picture. In this review we will describe and compare different imaging techniques for localizing inorganic as well as organic nanoparticles in tissues, cells and subcellular compartments. The visualization techniques include well-established methods, such as standard light, fluorescence, transmission electron and scanning electron microscopy as well as more recent developments, such as light and electron microscopic autoradiography, fluorescence lifetime imaging, spectral imaging and linear unmixing, superresolution structured illumination, Raman microspectroscopy and X-ray microscopy. Importantly, all methodologies described allow for the simultaneous visualization of nanoparticles and evaluation of cell and tissue changes that are of prime interest for toxicopathologic studies. However, the different approaches vary in terms of applicability for specific particles, sensitivity, optical resolution, technical requirements and thus availability, and effects of labeling on particle properties. Specific bottle necks of each technology are discussed in detail. Interpretation of particle localization data from any of these techniques should therefore respect their specific merits and limitations as no single approach combines all desired properties.

## Introduction

In the rapidly growing field of nanotechnology, recent developments have yielded a plethora of different nanoparticles (NP) with novel size-dependent properties that are very distinct from those of their bulk material [1–2]. On the one hand, the potential advantages of NP in biomedical research are manifold. For example, nanomaterials find application in surgical implants to improve tissue formation or due to their antibacterial action, they may be useful for gene or drug delivery systems as well as diagnostic imaging tools [3–7]. On the other hand, due to the entirely new properties of NP, the risk of adverse and even toxic side effects as well as accumulation of NP in the body has to be considered [1,5]. Clearly, the mentioning of all current medical applications, developments and future visions on NP technology is by far beyond the scope of this review and excellent review articles are available on NP pharmacology and toxicology in humans and animals [8–9]. However, despite all advancements in in vitro testing including permanent or primary cell lines and ex vivo organ cultures, the complexity of a living organism cannot be modeled in a test tube or culture dish. In this regard, similar to the assessment of effects of bulk material compounds, the microscopic assessment of expert pathologists is indispensable for the identification and characterization of target cells and structures as well as for the effects on and responses by these structures. For example, unfavorable tissue reactions directly induced by NP have to be monitored, such as degeneration and necrosis of target structures, NP-induced inflammation with influx and activation of immune cells, tissue fibrosis or even the induction of tumor growth [2,10–11]. Moreover, if the NP are destined for diagnostic or therapeutic applications in diseased tissue, the disease environment needs to be taken into account, either in terms of the effects of NP on the course of the disease or in terms of effects of the disease on the distribution and behavior of NP. For example, several studies have suggested an aggravation of allergic disease models following exposure with silica nanoparticles (SiO_2_-NP) [12–14]. Inorganic SiO_2_-NP hold great potential for several biomedical applications, including the selective targeting of cancer cells as well as drug or gene delivery systems due to their favorable biocompatibility and modification possibilities [15–16]. However, other authors have failed to see an aggravation of disease. In some cases, they even reported an alleviation of skin lesions following exposure with SiO_2_-NP or zinc oxide NP (ZnO-NP) [17–18]. ZnO-NP and titanium dioxide NP (TiO_2_-NP) are major ingredients of sunscreens [19] and their toxicity is of prime interest in the safety evaluation of NP. Importantly, for a precise understanding of the biological and toxicological effects of complex NP it is important to understand which part of the NP induces the observed effects, e.g., the inorganic core, the ligand shell, the protein corona or even the drug or label inside or associated with the particle. Therefore, more sophisticated imaging methods are needed that allow one to distinguish different parts of a NP within tissues or cells. Furthermore, the expertise of specially trained toxicological pathologists is essential in any multidisciplinary team involved in the development, characterization, and risk assessment of novel NP [20]. For this purpose, professional training and standardized certification of toxicological pathologists are firmly established, for example, by the European or American Colleges of Veterinary Pathologists [21–23] and specialization as well as continuing professional development are embedded in professional organizations such as the Society of Toxicologic Pathology (STP) in Europe, ESTP, or its US counterpart [21,24]. Today, the assessment of tissue and whole organisms through expert pathologists is essentially involved in the entire process of NP research and development, ultimately culminating in preclinical and clinical studies requested by regulatory authorities [25–27].

For a comprehensive understanding of the effects of NP in normal and diseased tissues, knowledge about their target structures, local and systemic NP distribution after administration and their final destination is indispensable. For an optimal pathological assessment, it is thus desirable to combine imaging techniques for the visualization of NP with techniques that are classically used for pathological examination of tissue sections from single organs and entire organisms. For this purpose, the light microscopic examination of approximately 5 µm thick sections of formalin-fixed and paraffin embedded (FFPE) tissue samples has proven indispensable for many decades [28–30]. However, many methodologies that have been used to localize or quantify NP, including elemental analyses by methods such as inductively coupled plasma atomic emission spectroscopy (ICP-AES) or atom absorption spectroscopy (AAS), require lysis, separation or homogenization of the cells and tissues [28,31–32]. The resulting consequence is a complete and irreversible loss of information about the association with or reactions by adjoining vital structures, making such approaches unfavorable for the purpose of concomitant pathological tissue assessment. Moreover, using antibody-based methods, the molecular phenotype in certain tissues can be interpreted within the biological context [33].

In this overview, we introduce and compare different imaging techniques for localizing inorganic NP like silica and iron oxide NP as well as organic NP such as polymer dendritic polyglycerol sulfates (dPGS) and chitosan NP. Importantly, all techniques described can be used for the simultaneous visualization of NP and toxicopathological assessment of the putative uptaking cells and adjacent tissues. All methodologies presented here should fulfill the following criteria: The techniques should be applicable for a pathologist in a more or less high-throughput manner, they should allow for a visualization of NP with concurrent evaluation of medically relevant tissue changes and they should be economic and practical for many samples. We describe well-established and widely used techniques, such as light, fluorescence, transmission electron and scanning electron microscopy which have already been reviewed by others from a different angle [20,34–35]. Furthermore, we will highlight and introduce new and more advanced techniques including light and electron microscopic autoradiography, fluorescence lifetime imaging, spectral imaging and linear unmixing, superresolution structured illumination, Raman microspectroscopy and X-ray microscopy.

## Review

### Light microscopy

Light microscopic examination has become the gold standard of pathologic evaluation and risk assessment of drugs and nanoparticles during the past 50 years. This technology uses sections of 3 to 8 µm thickness from FFPE or cryosectioned tissue samples, usually stained with hematoxylin and eosin (HE). As a required but well accepted simplification, two-dimensional (2D) images of a complex three-dimensional (3D) biologic structure are analyzed. Standardized trimming protocols help to reduce interregional variations and increase the reproducibility and comparability of toxicopathologic studies [25,36–38]. Light microscopy thus allows for a sensitive, efficient, and cost-effective evaluation of large amounts of microscopic information and largely satisfies demands by regulatory authorities [25]. In the past years, this technology has also proven useful in characterizing toxicological effects of various NP by histopathological examinations of large numbers of diverse tissues and organ systems [39–42].

**Figure 1:**
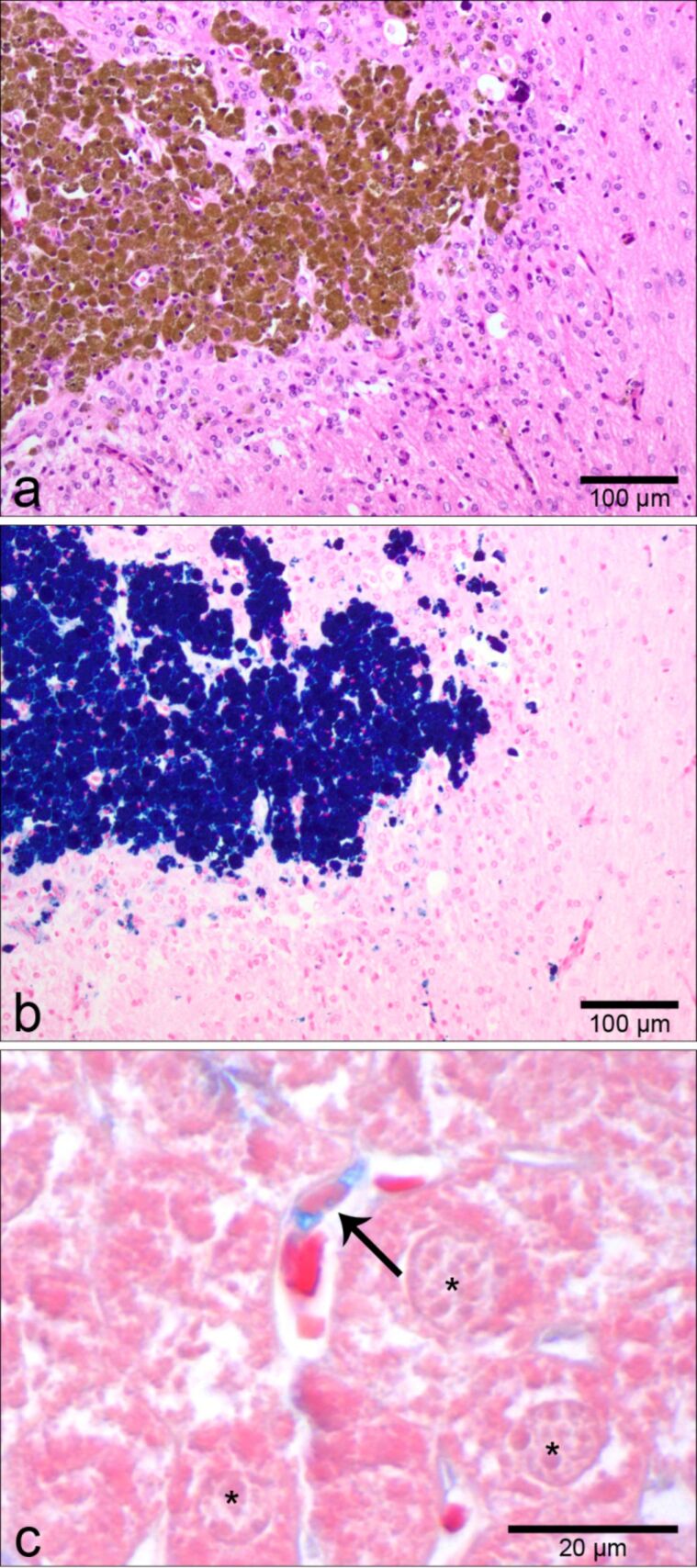
Nanoparticles may be detected through light microscopy by using chemical staining protocols that are conventionally employed in histopathology. For example, clusters of iron oxide nanoparticles can be visualized in HE-stained tissue sections as a finely granular brown material within the cells of a glioblastoma tumor (a, with kind approval of MagForce AG, Berlin, Germany). When serial sections from the same tissue were stained with a canonical stain for iron, Turnbull blue, the particles appear dark blue (b). As a second example, Alcian blue stain may be used to stain dendritic polyglycerol sulfates (dPGS) due to their negatively charged, sulfate rich shell. Organic dPGS amine accumulated in the cytoplasm of hepatic Kupffer cells (c, arrow). These liver specific macrophages are identified by their comma-shaped nuclei and their lining of hepatic sinusoids. Adjacent hepatocytes (c, asterisks) appear as light pink cells with finely stippled cytoplasm whereas erythrocytes within sinusoids can be identified by their intensely pink color.

The ideal setup would allow for NP detection and evaluation of surrounding tissue at the same time. Nevertheless, a certain magnification must be achieved for the detection of nanoparticles in the tissue due to their size of, by definition, less than 100 nm [20]. Single particles thus cannot be resolved due to the maximal diffraction limited resolution of 200 to 500 nm at highest light microscopic magnification. Thus, only NP that cluster or form aggregates of more than 200 nm in size in the tissue of interest can be visualized directly. In addition, these aggregates have to be able to attenuate visible light to be detectable by using light microscopy. For example, carbon nanotubes (CNT) used in medical or electronic devices that are known to induce granuloma formation and fibrosis in the lung following intratracheal exposure appear as grey structures when forming aggregates in tissue sections stained according to standard protocols [43–44]. Aggregated iron oxide nanoparticles are visible as brown deposits in HE-stained sections of glioblastomas (Figure 1a), a common brain tumor with high clinical relevance [45]. Such particles have similarly been visualized after targeting prostate cancer cells in humans [46]. Iron oxide nanoparticles have been introduced as diagnostic tool or for the treatment of various cancers [45–48]. In several applications, they have proven to possess excellent tumor-targeting efficacy [49]. Likewise, titanium dioxide nanoparticles, essential components of sunscreens, were visualized as yellow-brown particles on superficial stratum corneum layers in HE-stained skin samples following topical application [29].

In addition, specific characteristics of some NP may be used to visualize them through so-called special stains that have been developed by pathologists over many decades. Aggregates of the above mentioned iron oxide NP, for example, may be stained by several special stains for iron (Figure 1b), including Turnbull blue and Prussian blue [45,48,50], which are usually used to label pigments containing biogenic iron, such as hemosiderin [51]. Alcian blue is a histologic stain for the detection of negatively charged sulfate groups that occur, for example, in mucins [52–53]. The organic dendritic polyglycerol sulfate (dPGS) NP possess a complex, branching structure of polyglycerol residues with such negatively charged sulfate groups in their shells [54]. Several studies have suggested promising therapeutic and diagnostic potential for dPGS, including anti-inflammatory effects with rather specific accumulation in inflamed tissues [55–57]. Due to their sulfate groups and the specific staining properties of Alcian blue, this method has been used successfully, for example, for the detection of dPGS amine accumulated in Kupffer cells in the liver of mice following intravenous injection (Figure 1c) [56,58]. As a third example of NP detectability in tissues, single-walled CNT labeled by covalent binding of colloidal gold can be visualized through light microscopy by using silver enhancement [59]. In this method, the colloidal gold serves as a nucleation core for metallic silver. The silver layer formed around the gold core increases the particle size dramatically and imparts a black color when viewed by light microscopy [60]. However, any labeling of NP may possess the risk of changing their physicochemical characteristics and their bioreactivity [20]. Accordingly, previous studies have shown that surface modifications by additional molecular labels may significantly influence the physical and chemical properties of NP, thereby also altering their behavior in cells and tissues as well as toxicologically relevant responses [35], an effect that will be discussed in more detail below.

### Light and electron microscopic autoradiography

**Figure 2:**
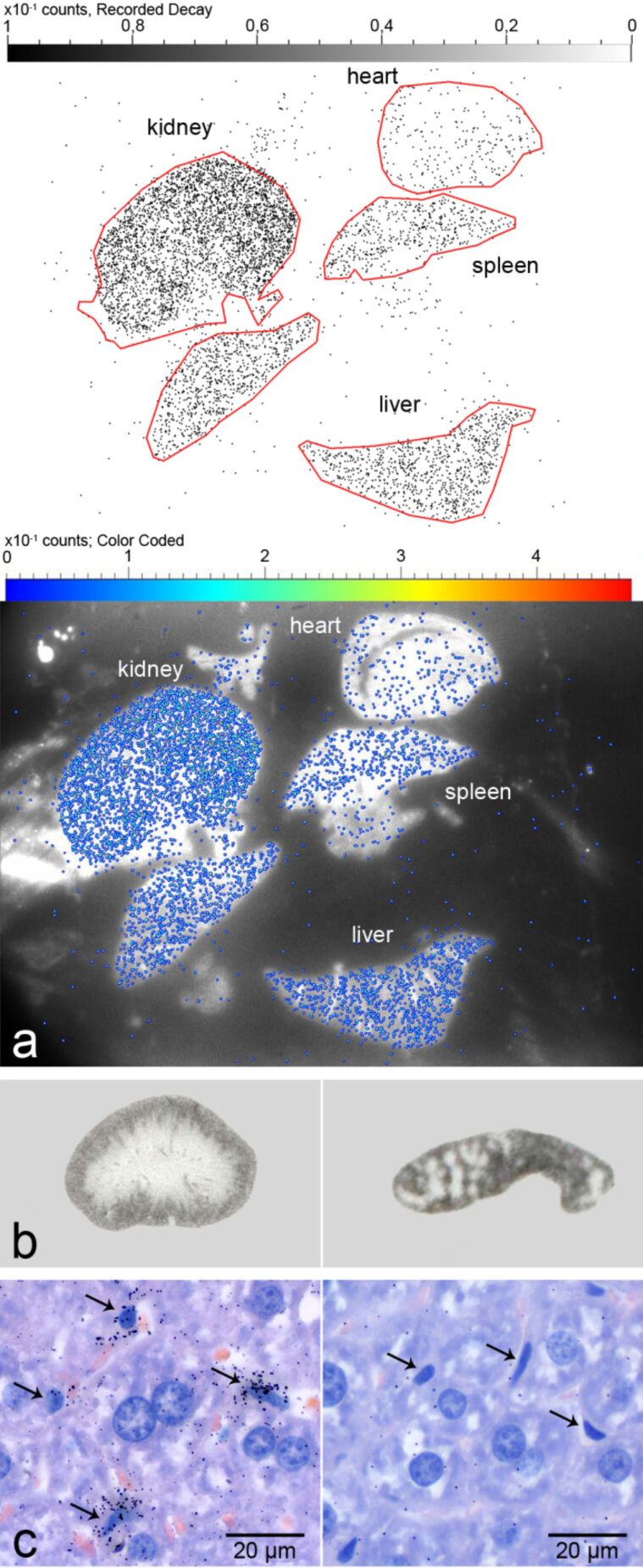
Autoradiographic detection of radiolabeled dPG^35^S amine in organs and tissues. (a) Semiquantitative micro imager analysis detected different amounts of dPG^35^S amine in mouse organs, decreasing from the kidney over the liver and the spleen to the heart one hour after intravenous injection (top panel: counting rates with regions of interest (indicated by red lines); bottom panel: combination of optical image and counting rates). (b) Single organ autoradiography of mouse kidney (left panel) and spleen sections (right panel) on X-ray film following dPG^35^S amine application identified distribution patterns of the radiolabeled NP in accordance with organ specific functional structure. In the kidney, NP were localized within the renal cortex (outer rim) whereas in the spleen they were clearly associated with the red pulp but not within lymphoid follicles (spared dots). (c) Light microscopic autoradiography with numerous radioactive decay-induced signals over Kupffer cells (arrows) in the liver of a mouse (left panel). Signals were sparse in adjacent hepatocytes with larger, more round nuclei. No signals were observed when unlabeled dPGS amine was used under otherwise identical experimental conditions (right panel). Hematoxylin and eosin-stained FFPE tissue sections.

Autoradiography is based on the spatiotemporal recording of radioisotopic decay in the context of surrounding tissues. It has been used to provide an overall picture of the systemic distribution of radiolabeled drugs or NP and even allows for a semiquantitative assessment of NP in tissues [61–62]. Various radioisotopes, including those of silver (^110m^Ag), carbon (^14^C) or indium (^111^I) have been used to label NP and to study their distribution throughout the whole body [63–65]. Radiation from these isotopes is usually detected and quantified by a gamma counter, micro imager, phosphoimager plates or autoradiographic films that are commonly used for X-ray exposures. However, such autoradiographs performed on cross sections of whole bodies or total organs of animals (Figure 2a and Figure 2b) [62–63,66–68] possess optical resolution limited to around one millimeter and fail to provide information on the cell or tissue levels [61].

In contrast, the optical resolution of light microscopic autoradiography (LMA) using photoemulsion-covered histological slides is limited by the optical resolving power of the light microscope (0.2 µm) and the grain size of the emulsion [69]. This technique has previously been used, for example, for the localization of specific nucleic acid sequence, e.g., chromosomes or viral infections, by in situ hybridization employing radiolabeled nucleic acid probes [70]. We reasoned that this approach could also be useful in detecting radiolabeled NP at light microscopic resolution and established a method for the visualization of dPG^35^S amine NP in pathohistologic slides (Figure 2c) [56,58]. The ^35^S-labeling of dPGS amine appears particularly suitable since the radioisotope replaces “cold” sulfur atoms in the outer shell of the NP, without changing its size, molecular weight or other biologically relevant physical or chemical properties [58]. Following intravenous application of dPG^35^S amine into mice, HE-stained FFPE tissue sections from various organs were covered with an autoradiographic emulsion. The slides were exposed for several days and finally fixed with a commercially available fixator for photographic films [56]. Autoradiography can also be adjusted for electron microscopy, EMA, by selecting emulsions with more appropriate grain diameter, tracking characteristics and sensitivities [71]. Both LMA and EMA offer high sensitivity due to the possibility of long exposure times with even small amounts of radioactive decay being detectable [56,58,72]. Importantly, after counterstaining the same slides with standard histostains, such as HE, routine pathohistologic examination of the same tissue is possible in direct context of the autoradiographic NP signals [56].

### Fluorescence microscopy

Conventional fluorescence microscopy is an essential tool in countless biomedical research applications and possesses a resolution similar to that of bright-field light microscopy [35,73]. As a consequence, NP can only be detected when they are closely clustered in aggregates or densely packed, for example, in phagocytic vacuoles. Pathologists routinely employ methods of fluorescent labeling for immunofluorescence, i.e., antibody-based identification of specific cell types, cell activation statuses, and apoptotic or degenerative changes [74]. In addition, fluorescence microscopy has been widely used in studies on the biodistribution of nanoparticles [28,48,75–78].

**Figure 3:**
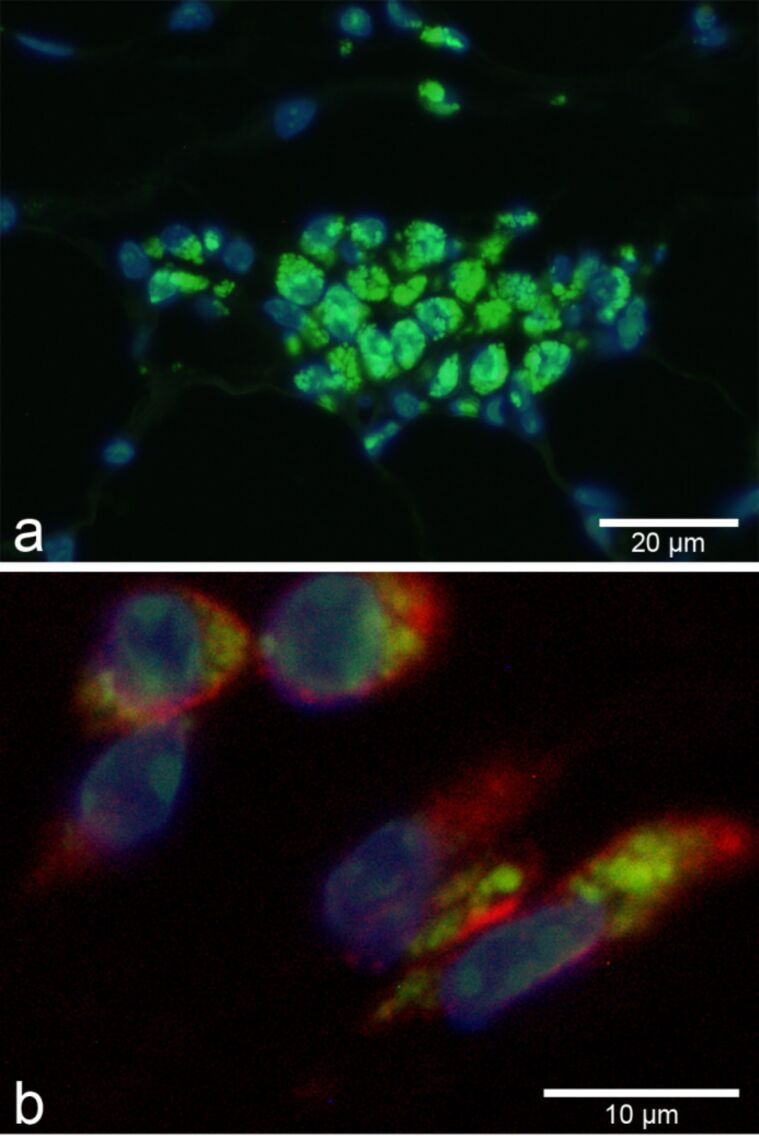
Aggregates of FITC-labeled SiO_2_-NP (green, 55 ± 6 nm in diameter) were visualized by fluorescence microscopy in macrophages following subcutaneous injection (a). Nuclei were counterstained with DAPI (blue). Subsequent immunofluorescent labeling (red) for the macrophage marker F4/80 identified macrophages as uptaking cells (b).

For fluorescence microscopic detection, NP are usually labeled with fluorescent dyes, such as fluorescein isothiocyanate (FITC) and indocarbocyanine. Several classes of NP have been localized after addition of such tags, including silica nanoparticles (SiO_2_-NP, Figure 3a) [28,75,79–81] and dPGS [82]. Inorganic SiO_2_-NP hold great potential for several biomedical applications, including the selective targeting of cancer cells as well as drug or gene delivery systems due to their favorable biocompatibility and modification possibilities [15–16]. However, labeling of NP always possesses the risk of changing their bioreactivity [20]. Thus, the site of labeling and the properties of the fluorochrome may have to be considered when predicting the altered physicochemical properties of the labeled NP, including its charge, size, molecular weight, and overall structure [35]. For example, integrating indocarbocyanine-3 with a molecular weight of 767 Da into nanoscaled macromolecules such as dPGS with a molecular weight of 1,300 Da [56] can be expected to drastically alter its physicochemical properties [56]. Consequently, if fluorochromes are integrated into the shell or bound to the surface of a NP, the properties of the new structure are hard to predict and may even alter its behavior in cells and tissues when compared to unlabeled particles [35]. In contrast, incorporation of the dye into the core of an otherwise unaltered surface of a NP may leave its overall properties unchanged, especially when an unlabeled shell can be grown around this core. In addition, the potential of additional specific surface functionalization and binding of other molecules of interest may remain completely similar to that of unlabeled particles [79,83–84].

Another disadvantage when using fluorescent dyes is their time-dependent photobleaching, which results in a more or less rapid fading of the fluorescent yield [85]. In this regard, the increased brightness and photostability of a fluorescent dye when incorporated into the core of SiO_2_-NP are particularly welcome [83]. Of note, some specifically engineered NP may hold inherent fluorescent properties, such as inorganic quantum dots (QD) [76]. QD used in bioapplications are typically composed of a semiconductor core (e.g., cadmium sulfide), an outer shell of a higher band semiconducting material (e.g., zinc sulfide) and a surface functionalization which may consist of various hydrophilic organic molecules including biomolecules or polymers [86]. QD with their size-dependent optical properties possess great potential as probe for biomedical imaging applications, as previously shown for the mapping of lymph node structures [87]. Their superior photostability, their broad excitation and narrow emission spectra offer additional advantages [85] that even allow for the detection of mixed QD populations with a single excitatory wavelength [86]. Furthermore, the impact of toxic ions released by QD on biological matter has been minimized by embedding QD into silica nanoparticles [88] without influencing the optical properties [89]. Unlike fluorescent dyes that undergo photobleaching, photoactivation is a remarkable property of QD enhancing their quantum yield over time [90].

Another group of highly photostable NP are lanthanide-doped upconversion NP (UCNP). Upconversion is an optical process in which the sequential absorption of two or more photons leads to the emission of light at shorter wavelength than the excitation wavelength [91]. UCNP feature a reduced cytotoxicity compared to QD and are, in contrast to fluorescent dyes or QD, excited by near infrared (NIR) light. By using long-wavelength NIR instead of ultra violet (UV) light, background autofluorescence typically caused by collagen and other autofluorescent structures of tissues is dramatically reduced [92]. NIR light penetrates deeper into biological tissue and thus in vivo tracking of UCNP holds promising applications [93]. The properties, synthesis as well as options of modifications and applications of UCNP have been reviewed recently [92–93]. Due to the low autofluorescence of tissues in NIR imaging, it has been successfully used for in vivo visualization of various other NP [94–95]. However, in vivo imaging may demonstrate the dynamic process of NP distribution but suffers from a poor spatial resolution [96]. Alternatively, high-resolution in vivo imaging of NP commonly requires more invasive methods [20,97].

One important drawback of fluorescence microscopy is the lack of visibility of other structures without fluorescent properties such as normal cells, membranes, and nuclei. The concurrent counterstain of nuclei with the blue fluorescent dye 4',6-diamidino-2-phenylindole (DAPI), for example, may overcome this disadvantage by allowing a view of all nucleated cells within the tissue. However, the selective counterstaining of nuclei only provides information on nuclear size, shape, and location which is insufficient for a complete histological assessment by a pathologist. Another approach is to compare and merge images of fluorescence microscopy and bright field microscopy by illuminating the same slide and location used for fluorescence microscopy with transmitted light [20,77].

As a further methodological option, fluorescent detection of NP can easily be combined with the immunofluorescent labeling of marker proteins which, for example, enable simultaneous identification of specific cell types, tumor markers, and even infectious agents [20]. In this combination, nanoparticles can even be spatially co-localized with marker proteins that help to identify both the uptaking cell type and its activation status (Figure 3b) [50,75,83]. Optical resolution of such co-localizations may be increased by various technical refinements, including confocal laser scanning microscopy which allows for serial 2D optical sectioning of the slide and even 3D reconstructions of complex tissues and single cells [98].

### Fluorescence lifetime imaging microscopy (FLIM)

Fluorescence lifetime imaging microscopy (FLIM) is a highly innovative and promising method which has been recently used in various biomedical and life science applications but not in routine toxicopathology so far. FLIM setups usually combine conventional laser scanning confocal microscopy with time-correlated single photon counting, thus, enabling the recording of fluorescence lifetime decay traces for each pixel. The fluorescence lifetime decay curve represents the excited-state decay behavior of a fluorochrome, usually decaying within the nanosecond range [99], and can be approximated by a single or a sum of several exponential functions [100–101]. A major advantage of FLIM is that the influence of the local environment of the fluorochrome can be monitored independently of the fluorochrome concentration [102].

**Figure 4:**
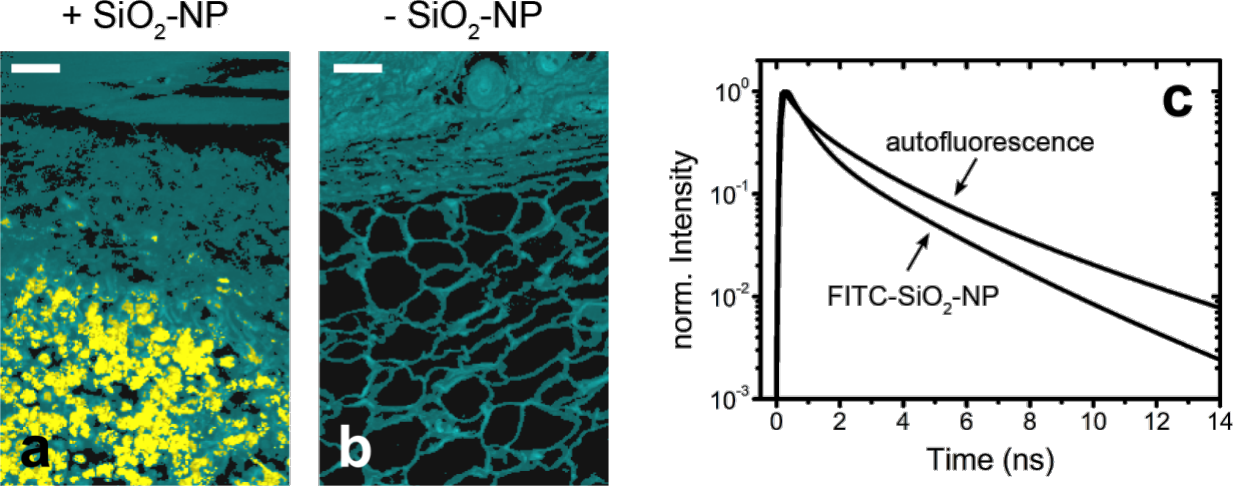
Discrimination of fluorescein isothiocyanate (FITC) labeled SiO_2_-NP (55 ± 6 nm in diameter) from the autofluorescent background of skin and subcutaneous tissue using fluorescence lifetime imaging microscopy (FLIM). False color coded sections of mouse skin including subcutis following subcutaneous particle injection from the site of injection (+SiO_2_-NP, (a)) and from the contralateral without NP (b) are presented. Cyan colored areas represent the autofluorescent background, while the yellow colored areas indicate a different lifetime species, which was identified as FITC-SiO_2_-NP. Scale bar = 50 µm. (c) Fluorescence lifetime curves of the tissue autofluorescence (mean lifetime curve) and of the FITC-SiO_2_-NP.

FLIM gains its information from the fluorescence decay curves and applications of this technique include environmental sensing of, amongst others, polarity, local pH, and calcium concentrations, as well as the study of protein interactions in living cells [102]. FLIM image analysis allows for a fast and reliable localization of target molecules, e.g., fluorochromes, against autofluorescent background [82,103]. FFPE tissue sample analysis in standard histopathologic examinations is often complicated by autofluorescence. The phenomenon of autofluorescence by endogenous fluorophores, e.g., NAD(P)H, collagen, melanin, and keratin [104] or due to tissue preparation artifacts may result in difficulties to distinguish a fluorescent signal yielded by fluorochromes from autofluorescence with conventional color cameras [105]. The discrimination of NP against the background has recently been shown for zinc oxide NP [106] as well as indocarbocyanine (ICC)-labeled core–multishell nanoparticles [107] in the skin, indocarbocyanine-labeled dPGS in the liver [82] and for subcutaneously injected silica-based NP (Figure 4) [81]. Recent developments of both hard- and software have further contributed to the advancement of FLIM [102] that seems to hold great potential for its use in a number of biomedical applications, for example, as diagnostic tool for histopathology [99].

### Spectral imaging and linear unmixing

Spectral imaging combined with linear unmixing is another technique that is not commonly used by pathologists to date but widely used in biomedical research. Similar to FLIM, it can be used on FFPE tissue sections to distinguish a fluorescent signal of fluorochromes from autofluorescence.

**Figure 5:**
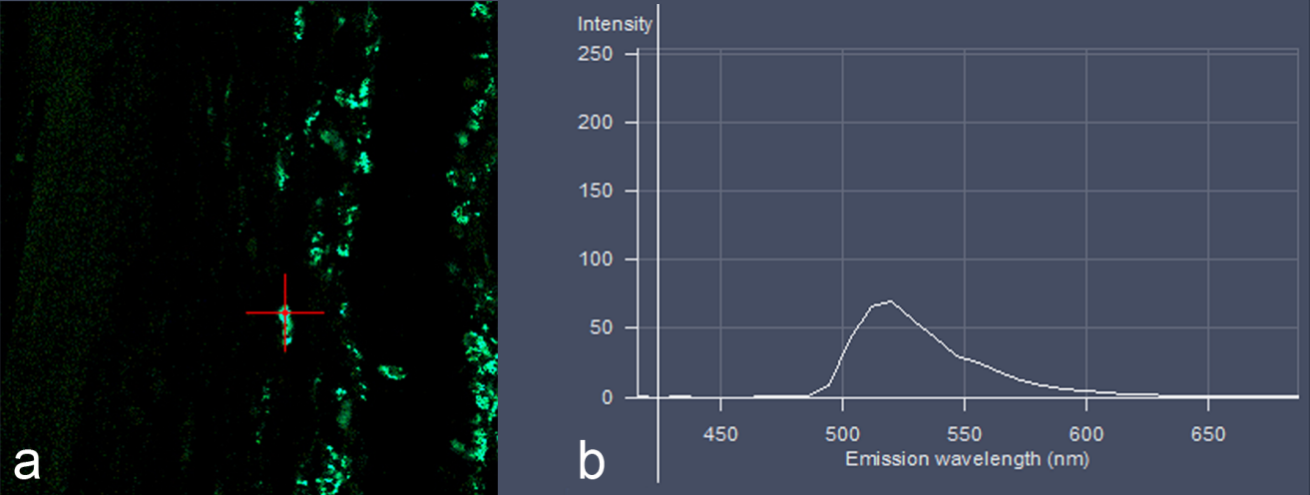
Spectral imaging and linear unmixing detection of green fluorescent SiO_2_-NP (55 ± 6 nm in diameter) in the subcutis of a mouse, indicated by the red cross (a), revealed an emission curve (b) which is highly specific for FITC with an emission maximum between 520 and 530 nm wavelength.

Whole emission spectra of the fluorescent signals are generated from a slide by using a multidetector array (Figure 5) [108]. In this approach, the generated emission spectrum of a certain dye used as label for NP differs unequivocally from the emission spectra generated by autofluorescent signals [109] and can be identified by using spectral libraries [33]. Moreover, this technique allows for the use of multiple fluorescent labels that cannot be distinguished in conventional fluorescence microscopic setups due to overlapping emission spectra [110]. Thus, multiple fluorescent labels may be used in a single slide to study interactions of cells and subcellular constituents in detail [110]. Spectral imaging and unmixing offers substantial improvement in signal-to-noise ratio and image contrast compared to the use of monochrome band-pass emission filters as employed in conventional fluorescence microscopes [111]. This approach also facilitates the detection of signals that are otherwise masked by autofluorescence [33]. For example, recent developments in the immunotherapy of tumor cells require a multimarker-based phenotyping and spectral imaging with subsequent unmixing appears to be valuable for the identification of tumor phenotypes within a single FFPE tissue slide [111–112]. Such data can even be analyzed automatically by using specialized software [112].

So far, spectral imaging with linear unmixing has only been used for the localization of QD as advanced fluorescent label with a narrow emission bandwidth [109,113]. However, for biodistribution and subcellular localization studies of fluorescently labeled NP, the spectral imaging and linear unmixing technique may become valuable in future work on the biodistribution of NP in the context of entire tissues. So far, one study reported the in vivo distribution of QD following various application routes and sites but more data are required [114].

Despite the huge potential of spectral imaging in the pathological examination, it would be desirable that advances in software and hardware as well as reagents will lead to a wider use of this technique in routine toxicopathologic examinations [33].

### Superresolution structured illumination microscopy (SR-SIM)

The optical resolution of fluorescence microscopy of approximately 200 nm has recently been increased twofold by superresolution structured illumination microscopy (SR-SIM) [115–116]. By rotating an optical grating and multiplication of the obtained pattern, so-called moiré fringes can be seen as stripes in the overlapping regions. These moiré fringes contain information about the unknown structure and the observer gains access to normally unresolvable information in the sample [115]. Thus, multiple images with different phases and orientations of the patterned light are recorded and reconstructed to obtain the SR-SIM image [117]. A detailed description on the principles of this technique was recently published elsewhere [118]. Moreover, the out-of-focus light is rejected computationally [119]. However, SR-SIM has not yet become a standard technique for histopathological examinations. It is primarily used for detailed analysis of subcellular structures, such as the cytoskeleton [115]. Importantly, this technique also allows for 3D reconstructions of information within the cell at a higher resolution level with all additional advantages described for confocal laser scanning microscopy [119–120].

**Figure 6:**
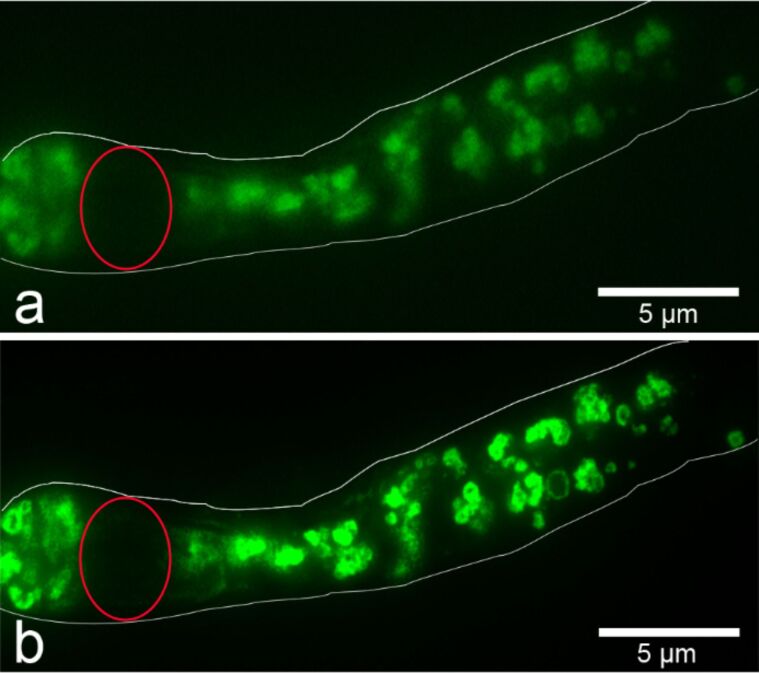
FITC-labeled SiO_2_-NP (55 ± 6 nm in diameter) within a single SiO_2_-containing cell of the subcutaneous tissue visualized by conventional widefield (a) versus superresolution structured illumination microscopy (b). Red circles indicate nuclei, white outlines indicate outer cell borders. FFPE subcutaneous tissue sections from mice following subcutanous injection of FITC-labeled SiO_2_-NP were sliced and dewaxed according to standard protocols. The dewaxed, unstained slides were covered and observed with the ELYRA inverted microscope (Carl Zeiss, Jena, Germany). The comparison of the conventional wide field image (a) and superresolution structured illumination image (b) yields a clearer and less blurry image of clustered FITC-labeled SiO_2_-NP.

Of note, conventionally prepared slides can be used for SR-SIM and no special preparation is required [121]. We used this technique to localize FITC-labeled SiO_2_-NP in FFPE tissue sections of mice that were sliced and dewaxed according to standard protocols. The dewaxed, unstained slides were covered with Roti-Mount FluorCare (Carl Roth GmbH, Karlsruhe, Germany) and examined with the ELYRA PS.1 inverted microscope combined with a confocal laser scanning microscope LSM 780 (both microscopes from Carl Zeiss, Jena, Germany). A z-stack over a distance of 4.49 µm consisting of 42 images was scanned on an area of 970 × 970 pixels. The images were subsequently processed by using a ZEN system 2012 (Carl Zeiss) and exported by using the ZEN lite software. When the images were compared with images obtained by wide-field fluorescence microscopy SR-SIM images were clearly superior due to higher resolution and less blurry appearance (Figure 6).

Of note, SR-SIM requires highly stable fluorochromes to achieve high quality images. Photobleaching of the fluorochrome after prolonged excitation time and high laser intensity necessary for SR-SIM may thus cause limitations in the reconstruction of the images [122–123]. However, the SR-SIM requires up to 10^6^-fold lower illumination intensities compared to single-molecule imaging or other superresolution approaches [119]. Other superresolution techniques including structured illumination have been used to study NP, such as QD due to their favored optical properties [122,124–125] and in investigations on the architecture of specific NP [117].

### Raman microspectroscopy

Raman microspectroscopy provides high-resolution imaging combined with chemical analysis without destruction of the biological sample and the use of labels [126–128]. Intrinsic chemical bond vibrations can be visualized and characterized combined with optical sectioning with diffraction-limited spatial resolution that can be approximated to 1 µm [129–130]. This technique has been used, for example, in in vivo and diagnostic imaging of various cancers in humans [131]. Margins between normal and pathological tissue can be determined based on biochemical spectra [132]. Furthermore, Raman microscopy can be used to track NP inside cells and at the same time provides information on biochemical interactions within the cell [126]. However, one disadvantage is that only low signal intensities are emitted by biomolecules themselves [132].

Advanced Raman techniques, such as surface-enhanced Raman spectroscopy (SERS), coherent anti-Stokes Raman spectroscopy (CARS), and stimulated Raman spectroscopy (SRS) have been used in the past along with microscopy approaches for studying biological matter along with nanoscopic systems [133–136]. Moreover, NP may be used as specific SERS-label which was coupled to the primary antibody for the immunohistochemical detection of proteins in tissue sections. Thus, it is possible to detect multiple proteins (up to 30 different labels) in the same tissue slide based on the different spectra yielded by each label and even a quantification of a target molecules becomes possible [137]. However, these more advanced Raman techniques require a careful preparation [132] but they are superior compared to conventional Raman approaches due to their enhanced sensitivity and selectivity.

Raman microspectroscopy enables the penetration into biological tissue up to several hundred microns in depth and CARS, for example, has been successfully used to track metal oxide and deuterated quaternary ammonium palmitoyl glycol chitosan (dGCPQ) nanoparticles in fish and mice, respectively [127,138]. Raman microscopy provides a valuable tool for future applications in toxicopathological evaluation of NP due to the independence of additional labels, the ease of quantification of NP and the wide applicability on unstained tissue slides as well as for bulk samples with no or only minimal sample preparation [132,139]. However, Raman microspectroscopy has several drawbacks. First, it is challenging to interpret the complex and overlapping bands of Raman spectroscopy into meaningful, biological information [140]. Another limitation is that individual NP cannot be identified but they can only be localized in their cellular context [138] due to the limited spatial resolution of about 1 µm [130]. A further disadvantage is the long time required for the acquisition of images covering larger tissue areas [141]. For example, a typical scan area of Raman microspectrometry is quite small with about 300 × 100 µm in size. In order to acquire images with thousands of spectra, it takes several hours to expose such a sample [132]. Moreover, weak Raman signals may be overwhelmed by stronger autofluorescent signals from the tissue sample itself. This can be reduced by using NIR excitation light but the speed of data acquisition is reduced and weak signals may be lost due to a reduced sensitivity of NIR signals [141]. For a wider application of label-free Raman microspectroscopy, it is desirable to shorten the image acquisition. However, enhanced signal intensities would be required for this [132]. To address these issues, more advanced Raman techniques, including CARS, SERS and SRS have been developed [140]. CARS imaging is faster compared to the spontaneous Raman microspectroscopy but special lasers as well as further processing tools are required to translate the CARS spectrum to a Raman spectrum due to the more complex CARS spectra. However, as a drawback of CARS spectroscopy, a non-resonant background is usually present [141]. SRS overcomes the latter two limitations of CARS [140]. Furthermore, the accessibility of biological samples for CARS is also limited due to high laser powers that might destroy the sample as well as a high concentration of certain molecules that are required [141].

### Soft X-ray microscopy and spectromicroscopy

Soft X-ray microscopy techniques combine high spatial resolution in the few-nanometer range with chemical selectivity by specific excitation processes and deep penetration into tissues. A further advantage is that aqueous samples such as tissues can be used without previous chemical fixation or other pretreatment. Most frequently used is the so called water window above the carbon K-edge and below the O K-edge in which C and N strongly absorb, whereas water is almost transparent. Consequently, organic materials have an excellent contrast within this range [142–143]. These requirements can be fulfilled by full-field transmission X-ray microscopic techniques in the soft regime and even tomographic analyses of biological samples of up to 10 µm thickness are possible [143]. Besides high contrast and penetration depth, synchrotron radiation in the soft X-ray regime may be tuned for spectromicroscopy and chemical identification of the X-ray absorbing elements in biological samples [143]. Soft X-ray spectromicroscopy techniques have been used for probing protein interactions with model biomaterial surface [142] and various quantitative analyses. At the C 1s edge the detection limit of this technique is in the part per thousand range [142] with a spatial resolution down to 10 nm [144].

The various capabilities of full-field transmission X-ray microscopy (TXM) include 3D tomography, quantification of absorption, and chemical identification through X-ray fluorescence and X-ray absorption near edge structure imaging [143]. Gold NP were visualized in mice by using full-field high-resolution transmission X-ray microscopy combined with a potassium permanganate staining of FFPE-tissue sections of the cerebellum and the liver [145]. Soft X-ray microscopy has been successfully applied for 3D imaging of vitrified cells without any further staining [146]. That study identified subcellular compartments in adenocarcinoma cells in a significantly faster and less laborious manner compared to 3D cryo-electron tomography [146]. In addition, this approach may also be applied for plunge-vitrified tissue in the future.

**Figure 7:**
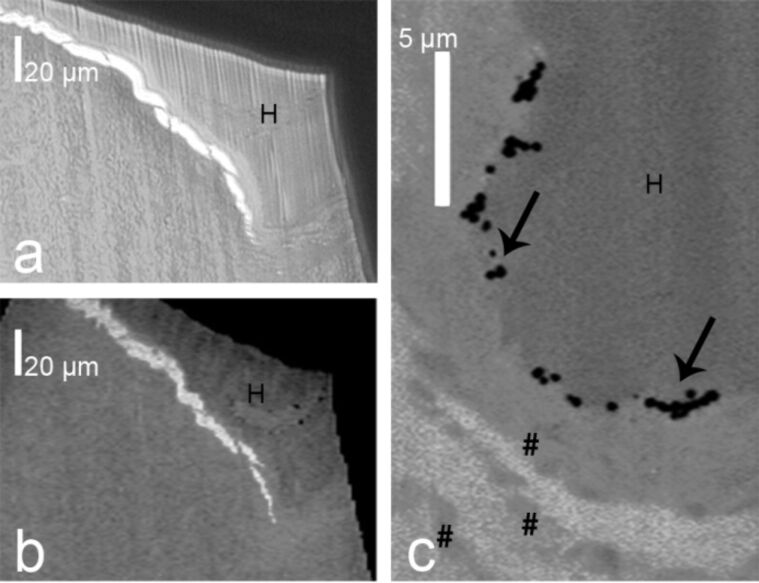
Light microscopy image (a) and scanning transmission X-ray microscopy (STXM) image (b) of a hair follicle opening with a central hair (H) at 270 eV of a 350 nm ultramicrotome section of human facial skin incubated with silica particles (161 ± 13 nm) with a 42 ± 3 nm gold core. Higher magnification of the STXM image visualized single particles (arrows) in an infundibulum on the surface and between corneocytes (pound sign) of the stratum corneum (c).

High-brilliance synchrotron radiation is tightly focused on a sample in scanning transmission X-ray microscopy (STXM) [147–149]. The sample is then raster-scanned while the intensity of the transmitted X-rays is recorded, thus, a 2D image is obtained. STXM has been used, for example, for studying cells in vitro [144,150]. We used STXM for a penetration study on gold core particles with silica shells with two sizes as well as silica particles with a gold shell in excised human skin (Figure 7) [151]. Following topical particle application, ultramicrotome sections of these samples were analyzed with light microscopy and STXM (Figure 7). High resolution STXM image analysis revealed single particles within the superficial layer of the stratum corneum [151].

A combination of STXM with X-ray fluorescence (XRF) microprobe has been used to study the fate of zinc oxide nanoparticles in vitro. Thereby, microfocused XRF elemental mapping yielded the local distribution of zinc in the cells and micro-X-ray absorption near-edge structure (XANES) spectroscopy allowed for the identification of different zinc species present in the sample [144]. Others used STXM and compact source transmission X-ray microscopy for subcellular imaging of vascular smooth muscle cells and characterized the local calcium distribution by using spectromicroscopy at the calcium L_3,2_ edges [150].

Despite the advantages of soft X-ray microscopy compared with fluorescence or electron microscopy techniques, it has only been applied for few biomedical samples so far due to the small number of synchrotron radiation facilities worldwide.

### Transmission electron microscopy (TEM)

To use an electron beam transmitting a sample can provide significantly higher spatial resolution (down to 0.1 nm) and higher magnifications by transmission electron microscopy compared to all light microscopic methods described above. On the one hand, TEM is still an important technique in the toxicological assessment by pathologists. It provides detailed information on subcellular structures regarding potentially toxicological changes, for example, changes in size, structure or number of cellular organelles highly responsive to all stress [152]. On the other hand, the sample preparation is more laborious and prone to artifacts compared to other microscopic methods [152–153]. The time intensive tissue preparation and analysis limit the analytical throughput of samples and result in a small volume of 1 to 10 µm^3^ that is usually analyzed [20,29,35,153–154]. Scanning transmission electron microscopy (STEM) has been presented recently as an elegant approach to overcome this limitation [154]. The authors simultaneously recorded bright and dark field STEM images of gold NP in murine liver tissue using optimized contrast setting and thus were able to analyze 243,000 μm^3^ of liver tissue in a single setting [154].

Furthermore, the image interpretation of TEM is more challenging than that of light microscopic techniques [20,152]. Tissue responses to NP, such as inflammation, fibrosis or necrosis [39,43,155] and endogenous structures, such as collagen fibers or immune cell granules must be distinguished from NP, requiring a deep understanding of normal and diseased cellular ultrastructure which is the typical domain of a pathologist [20].

**Figure 8:**
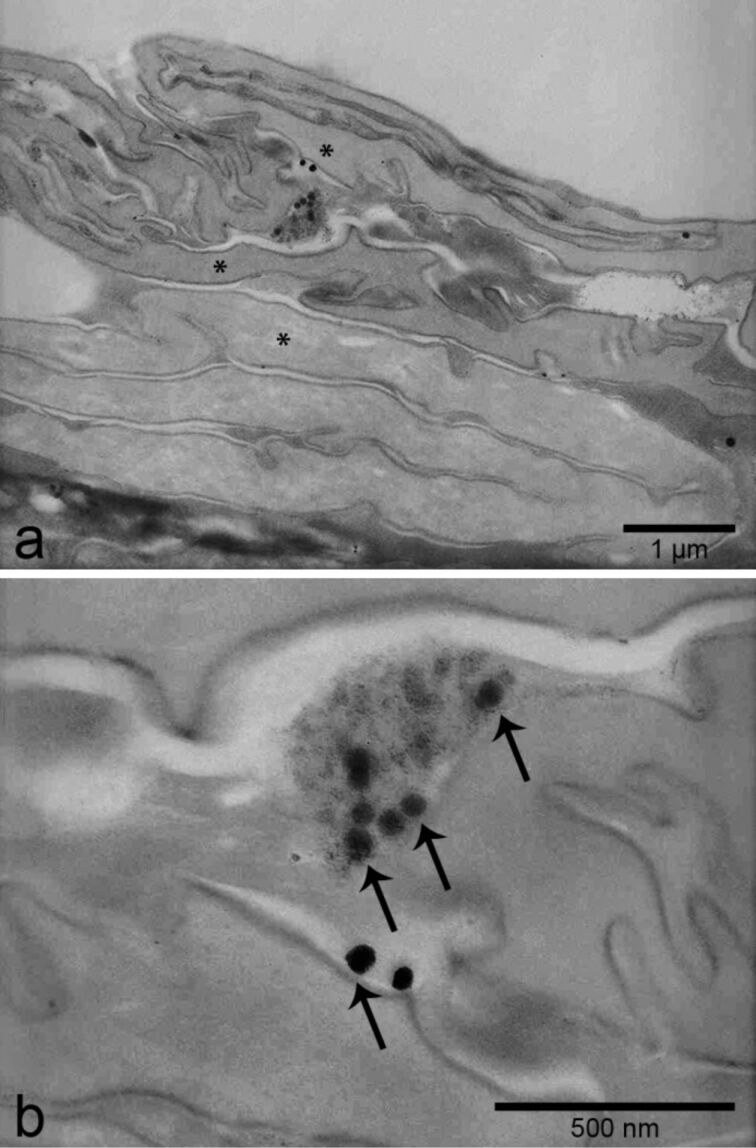
Transmission electron microscopic detection of single electron-dense SiO_2_-NP (55 ± 6 nm in diameter) between corneocytes (asterisks) of the mouse skin (a). Higher magnification of the same region (b) reveals single spherical nanoparticles (arrows). Sections were prepared without uranyl acetate and lead citrate staining.

Due to its high resolution, TEM is typically employed to visualize single NP (Figure 8). However, a certain electron density of the NP is required for this purpose. For example, organic NP provide only low contrast in tissues, so that in general other techniques are necessary to study these [56]. For the visualization of electron-dense inorganic NP, for example, titanium dioxide, SiO_2_-NP or QD, TEM has been widely used to characterize the morphology and size of NP as well as their location in tissues [28,35,39,113,156–158]. It has to be kept in mind, however, that artifacts due to staining with lead citrate and uranyl acetate can easily be misinterpreted as NP. To avoid or at least reduce this risk, some authors preferred not to stain the sample or used uranyl acetate only [29,159]. NP with a low electron density, such as Raman-active-SiO_2_-NP or CNT, can be labeled, for example, by synthesizing NP around a gold core [154] or covalent binding of colloidal gold prior to their employment in vivo [59] which increases their visibility in TEM by enhanced contrast.

As a well-known artifact, the cell volume shrinks dramatically during the standard preparation of electron microscopy samples, mostly due to chemical fixation [160–161]. In addition, dehydration effects may deleteriously affect the ultrastructure of tissues [162]. Therefore, cryogenic approaches in which chemical fixation is avoided and the normal hydration state is maintained may be advantageous for a more life-like preservation of biological samples [163]. Cryo-TEM has been used to study skin on the ultrastructural level [164], to characterize NP [165] and for localizing NP in vitro [166].

As an additional technical refinement, qualitative elemental analysis techniques can be performed on biological samples following NP exposure by coupling a microanalysis system to the electron microscope [35]. Energy dispersive X-ray (EDX) analysis and electron energy loss spectroscopy (EELS) allow one to identify the elemental composition of a sample [167–168]. An elemental analysis may be of prime interest to distinguish NP in the tissue from artifacts that may be produced by staining procedures [35]. EDX analysis and EELS can be combined with TEM as well as scanning electron microscopy (SEM) and have been used for the detection of, for example, titanium dioxide NP, QD, and silver NP [167,169–172]. In addition, a combination of EDX analysis with a scanning method (STEM or SEM) allows for 2D-mapping of tissues or cells. As a result, the distribution of specific elements such as phosphorus, calcium and iron can be identified [173] and conclusions about toxic effects induced by NP may be drawn.

In summary, TEM is thus regarded as a useful addition to a series of microscopic tools, rather than a first-choice high-throughput imaging technique for NP in tissues [20].

### Scanning electron microscopy (SEM)

Imaging through scanning electron microscopy (SEM) is based on a raster scan of an electron beam over a surface and the detection of the backscattered electrons and the ejected secondary electrons [174]. SEM can be combined with a transmission electron detector for STEM or analytical methods, for example, EDX or EELS as described for TEM. For example, SEM with EDX analysis has been used to study the fate of silver NP and ions in an in vitro human gastrointestinal digestion model [175]. Chemically or cryo-fixed tissue sections are often sputter-coated, i.e., covered with a thin layer of conductive material, for example, gold [176], to enhance the electrical conductivity of the specimen [177]. As an alternative, no coating of the specimen is required when working with low-voltage SEM setups [178]. High resolution SEM with a spatial resolution down to 1.3 nm [179] provides 3D images of nanoscale materials within biological specimens [20]. Interactions between NP and the surrounding cells can be well visualized and have been demonstrated, for example, in lung sections following NP exposure [34]. Here, it was possible to detect single multi-walled CNT within alveolar macrophages and even when penetrating through the mesothelial surface of the pleura following inhalation in mice [180].

Chemical tissue fixation for SEM may have the same unfavorable shrinkage effects on cells and tissues as described for TEM. To overcome this problem, a combination of fast-freezing techniques such as high-pressure and plunge freezing has been used for the ultrastructural examination of skin and other organs without such artifacts [161,181–182]. In addition, cryo-SEM has also been used for the concomitant localization of NP in cells [183].

## Conclusion

A toxicopathological evaluation of tissues is indispensable for any risk assessment and hazard identification of NP [20]. Each of the microscopic techniques reviewed here has its advantages and limitations, as summarized in Table 1.

**Table 1:** Comparison of analytical methods for the visualization of nanoparticles in the context of adjacent tissues.

method	need for labeling	major advantages	major disadvantages

light microscopy in FFPE tissues	depends on NP used	easy, low cost, excellent evaluation of pathomorpholgic effects in context of NP detection	limited resolution (above 200 nm), only for few NP species available as imaging technique, staining artifacts, specificity of staining protocols
light and electron microscopic autoradiography	yes	highly sensitive and specific, excellent evaluation of pathomorphologic changes	long exposure time of the sample, expensive, radioactive labeling, radiation safety requirements
fluorescence microscopy	yes, except for QD and UCNP	easy, low cost, immunofluorescent identification of target cells and subcellular compartments possible, high specificity	limited resolution (above 200 nm), evaluation of pathomorphologic changes impossible without immunolabeling of cells, autofluorescence
fluorescence lifetime imaging microscopy	yes, except for QD and UCNP	high selectivity, differentiation of fluorescent NP from autofluorescence, immunofluorescent labeling of cells and compartments possible with high numbers of different markers on one slide	limited resolution (above 200 nm)
spectral unmixing	yes, except for QD and UCNP	easy, immunofluorescent labeling of cells and compartments possible with high numbers of different markers on one slide, high specificity, differentiation of fluorescent NP from autofluorescence	limited resolution (above 200 nm)
superresolution structured illumination	yes, except for QD and UCNP	easy, immunofluorescent identification of target cells and subcellular compartments possible, high selectivity, improved resolution (~100 nm), 3D reconstructions possible	autofluorescence, photobleaching of fluorochrome
Raman microspectral imaging	no	highly selective for chemical bonds within tissue and cells, interactions of cells and NP including chemical changes can be studied, no or minimal sample preparation required	limited resolution (1 µm), time consuming imaging process, challenging spectra analysis, autofluorescence
scanning transmission X-ray microscopy	depends on X-ray absorption contrast relative to tissue, sufficient for most inorganic NP	increased resolution compared to fluorescence microscopy, high spatial resolution (≥10 nm), element and site specific method, no staining necessary, in situ evaluation of changes of chemical composition of NP, imaging of fully hydrated samples of up to 10 µm thickness possible, correlated imaging with CLSM and other light microscopic techniques possible	significant technical effort required, limited number of expertimental facilities, limited evaluation of pathomorphological changes
cryo-3D-X-ray microscopy	depends on X-ray absorption contrast relative to tissue, sufficient for most inorganic NP	high resolution (≥20 nm), 3D imaging, evaluation of complete shock-frozen cells and thick tissues samples, no changes of cells and tissue due to embedding, slicing and contrasting	technically demanding, limited number of experimental facilities
transmission electron microscopy including cryo-TEM	depends on electron density	high resolution (down to 0.1 nm); detailed information on subcellular changes and NP structure, visualization of single NP, combination with EDX or EELS allows for elemental analysis within sample	only for electron-dense NP, time intensive sample preparation and analysis, staining and shrinking artifacts (no shrinking in cryo-TEM), only thin tissue sections (70 nm) can be studied
scanning electron microscopy including cryo-SEM	depends on electron density	high resolution (down to 1 nm), detailed information of NP–cell interactions, combination with EDX or EELS allows for elemental analysis within sample	complex sample preparation, technically demanding

A single technique is often insufficient to address all questions regarding the distribution of NP within the body, the cellular uptake, and the target cells and organs. But a combination of different detection methods may provide reliable information on the NP biodistribution and associated histomorphologic changes. Most of the techniques fail to detect single NP due to limited resolution and thus may possess limited sensitivity. Instead, many of these techniques may be more suitable for toxicopathologic evaluations of the tissues.

Importantly, due to their limited resolution, the majority of imaging techniques preferably used for localizing NP in tissue context do not allow for an exact quantitative determination of the number of nanoparticles in the tissues investigated. As a simple approach, semiquantitative estimation of NP numbers in tissues can be achieved by counting fluorescent spots or through a pixel analysis [184–185]. For a precise quantification of NP, however, spectroscopic or scintillation analyses may be employed, as recently demonstrated [71]. For example, while the authors failed to detect SiO_2_-NP in the liver and spleen by fluorescence microscopy and TEM, elemental analysis by inductively coupled plasma optical emission spectrometry (ICP-OES) revealed elevated silica content in the organs compared to a control group [28].

Taken together, for virtually all techniques described here, a compromise has to be made between optical resolution and thus sensitivity of NP detection and suitability for toxicological evaluation in a larger tissue context. The advantages and disadvantages of each detection method must be weighed against each other with regard to the study goal, nature of particles to be detected, need for reliable histopathology, technical expertise and equipment available.

## References

[1] De Jong W H, Borm P J A (2008). Int J Nanomed.

[2] Fischer H C, Chan W C W (2007). Curr Opin Biotechnol.

[3] Ballarre J, Desimone P M, Chorro M, Baca M, Orellano J C, Ceré S M (2013). J Struct Biol.

[4] Depan D, Misra R D K (2014). Mater Sci Eng, C.

[5] Fadeel B, Garcia-Bennett A E (2010). Adv Drug Delivery Rev.

[6] Dohan Ehrenfest D M, Vazquez L, Park Y-J, Sammartino G, Bernard J-P (2011). J Oral Implantol.

[7] Ryman-Rasmussen J P, Riviere J E, Monteiro-Riviere N A (2006). Toxicol Sci.

[8] Matoba T, Egashira K (2014). Int Heart J.

[9] Underwood C, van Eps A W (2012). Vet J.

[10] Ayres J G, Borm P, Cassee F R, Castranova V, Donaldson K, Ghio A, Harrison R M, Hider R, Kelly F, Kooter I M (2008). Inhalation Toxicol.

[11] Sayes C M, Warheit D B (2009). Wiley Interdiscip Rev: Nanomed Nanobiotechnol.

[12] Brandenberger C, Rowley N L, Jackson-Humbles D N, Zhang Q, Bramble L A, Lewandowski R P, Wagner J G, Chen W, Kaplan B L, Kaminski N E (2013). Part Fibre Toxicol.

[13] Hirai T, Yoshikawa T, Nabeshi H, Yoshida T, Tochigi S, Ichihashi K-i, Uji M, Akase T, Nagano K, Abe Y (2012). Part Fibre Toxicol.

[14] Hirai T, Yoshikawa T, Yoshida T, Ichihashi K-i, Takahashi H, Nabeshi H, Yoshioka Y, Tsutsumi Y (2012). Toxicol Lett.

[15] Wang L, Zhao W, Tan W (2008). Nano Res.

[16] Knopp D, Tang D, Niessner R (2009). Anal Chim Acta.

[17] Ostrowski A, Nordmeyer D, Mundhenk L, Fluhr J W, Lademann J, Graf C, Rühl E, Gruber A D (2014). Nanoscale Res Lett.

[18] Ilves M, Palomäki J, Vippola M, Lehto M, Savolainen K, Savinko T, Alenius H (2014). Part Fibre Toxicol.

[19] Papakostas D, Rancan F, Sterry W, Blume-Peytavi U, Vogt A (2011). Arch Dermatol Res.

[20] Hubbs A F, Sargent L M, Porter D W, Sager T M, Chen B T, Frazer D G, Castranova V, Sriram K, Nurkiewicz T R, Reynolds S H (2013). Toxicol Pathol.

[21] Ettlin R A, Bolon B, Pyrah I, Konishi Y, Black H E (2008). Exp Toxicol Pathol.

[22] (2014). ECVP European College of Veterinary Pathologists.

[23] (2014). ACVP American College of Veterinary Pathologists.

[24] Ruehl-Fehlert C, Bradley A, George C, Germann P-G, Provencher Bolliger A, Schulte A (2005). Exp Toxicol Pathol.

[25] Crissman J W, Goodman D G, Hildebrandt P K, Maronpot R R, Prater D A, Riley J H, Seaman W J, Thake D C (2004). Toxicol Pathol.

[26] Sadrieh N (2014). FDA considerations for regulation of nanomaterial containing products.

[27] Kramer J A, Sagartz J E, Morris D L (2007). Nat Rev Drug Discovery.

[28] Fu C, Liu T, Li L, Liu H, Chen D, Tang F (2013). Biomaterials.

[29] Adachi K, Yamada N, Yamamoto K, Yoshida Y, Yamamoto O (2010). Nanotoxicology.

[30] Cho W-S, Choi M, Han B S, Cho M, Oh J, Park K, Kim S J, Kim S H, Jeong J (2007). Toxicol Lett.

[31] Rancan F, Gao Q, Graf C, Troppens S, Hadam S, Hackbarth S, Kembuan C, Blume-Peytavi U, Rühl E, Lademann J (2012). ACS Nano.

[32] Vogt A, Combadiere B, Hadam S, Stieler K M, Lademann J, Schaefer H, Autran B, Sterry W, Blume-Peytavi U (2006). J Invest Dermatol.

[33] Levenson R, Beechem J, McNamara G (2012). Anal Cell Pathol.

[34] Hubbs A F, Mercer R R, Benkovic S A, Harkema J, Sriram K, Schwegler-Berry D, Goravanahally M P, Nurkiewicz T R, Castranova V, Sargent L M (2011). Toxicol Pathol.

[35] Marquis B J, Love S A, Braun K L, Haynes C L (2009). Analyst.

[36] Kittel B, Ruehl-Fehlert C, Morawietz G, Klapwijk J, Elwell M R, Lenz B, O'Sullivan M G, Roth D R, Wadsworth P F (2004). Exp Toxicol Pathol.

[37] Morawietz G, Ruehl-Fehlert C, Kittel B, Bube A, Keane K, Halm S, Heuser A, Hellmann J (2004). Exp Toxicol Pathol.

[38] Ruehl-Fehlert C, Kittel B, Morawietz G, Deslex P, Keenan C, Mahrt C R, Nolte T, Robinson M, Stuart B P, Deschl U (2003). Exp Toxicol Pathol.

[39] Wu J, Liu W, Xue C, Zhou S, Lan F, Bi L, Xu H, Yang X, Zeng F-D (2009). Toxicol Lett.

[40] Nishimori H, Kondoh M, Isoda K, Tsunoda S-i, Tsutsumi Y, Yagi K (2009). Eur J Pharm Biopharm.

[41] Nishimori H, Kondoh M, Isoda K, Tsunoda S-i, Tsutsumi Y, Yagi K (2009). Eur J Pharm Biopharm.

[42] Isoda K, Hasezaki T, Kondoh M, Tsutsumi Y, Yagi K (2011). Pharmazie.

[43] Shvedova A A, Kisin E R, Mercer R, Murray A R, Johnson V J, Potapovich A I, Tyurina Y Y, Gorelik O, Arepalli S, Schwegler-Berry D (2005). Am J Physiol: Lung Cell Mol Physiol.

[44] Porter D W, Hubbs A F, Mercer R R, Wu N, Wolfarth M G, Sriram K, Leonard S, Battelli L, Schwegler-Berry D, Friend S (2010). Toxicology.

[45] van Landeghem F K H, Maier-Hauff K, Jordan A, Hoffmann K-T, Gneveckow U, Scholz R, Thiesen B, Brück W, von Deimling A (2009). Biomaterials.

[46] Johannsen M, Gneveckow U, Taymoorian K, Thiesen B, Waldöfner N, Scholz R, Jung K, Jordan A, Wust P, Loening S A (2007). Int J Hyperthermia.

[47] Jordan A, Scholz R, Maier-Hauff K, van Landeghem F H, Waldoefner N, Teichgraeber U, Pinkernelle J, Bruhn H, Neumann F, Thiesen B (2006). J Neuro-Oncol.

[48] Stelter L, Pinkernelle J, Michel R, Schwartländer R, Raschzok N, Morgul M, Koch M, Denecke T, Ruf J, Bäumler H (2010). Mol Imaging Biol.

[49] Santhosh P B, Ulrih N P (2013). Cancer Lett.

[50] Bumb A, Regino C A S, Egen J G, Bernardo M, Dobson P J, Germain R N, Choyke P L, Brechbiel M W (2011). Mol Imaging Biol.

[51] Bunting H (1949). Biotech Histochem.

[52] Lev R, Spicer S S (1964). J Histochem Cytochem.

[53] Dorofeyev A E, Vasilenko I V, Rassokhina O A, Kondratiuk R B (2013). Gastroenterol Res Pract.

[54] Türk H, Haag R, Alban S (2004). Bioconjugate Chem.

[55] Dernedde J, Rausch A, Weinhart M, Enders S, Tauber R, Licha K, Schirner M, Zügel U, von Bonin A, Haag R (2010). Proc Natl Acad Sci U S A.

[56] Holzhausen C, Gröger D, Mundhenk L, Welker P, Haag R, Gruber A D (2013). Nanomedicine.

[57] Biffi S, Dal Monego S, Dullin C, Garrovo C, Bosnjak B, Licha K, Welker P, Epstein M M, Alves F (2013). PLoS One.

[58] Gröger D, Paulus F, Licha K, Welker P, Weinhart M, Holzhausen C, Mundhenk L, Gruber A D, Abram U, Haag R (2013). Bioconjugate Chem.

[59] Mercer R R, Scabilloni J, Wang L, Kisin E, Murray A R, Schwegler-Berry D, Shvedova A A, Castranova V (2008). Am J Physiol: Lung Cell Mol Physiol.

[60] Oliver C, Oliver C, Jamur M C (2010). Use of Immunogold with Silver Enhancement. Immunocytochemical Methods and Protocols.

[61] Stojanov K, Zuhorn I S, Dierckx R A J O, de Vries E F J (2012). Pharm Res.

[62] Huang F-Y, Lee T-W, Kao C-H K, Chang C-H, Zhang X, Lee W-Y, Chen W-J, Wang S-C, Lo J-M (2011). Cancer Biother Radiopharm.

[63] Al-Sid-Cheikh M, Rouleau C, Pelletier E (2013). Mar Environ Res.

[64] Al-Hallak M H D K, Sarfraz M K, Azarmi S, Roa W H, Finlay W H, Rouleau C, Löbenberg R (2012). Ther Delivery.

[65] Lee W-C, Hwang J-J, Tseng Y-L, Wang H-E, Chang Y-F, Lu Y-C, Ting G, Whang-Peng J, Wang S-J (2006). Nucl Instrum Methods Phys Res, Sect A.

[66] Sakamoto A, Ido T (1993). Brain Res.

[67] Dassler K, Roohi F, Lohrke J, Ide A, Remmele S, Hütter J, Pietsch H, Pison U, Schütz G (2012). Invest Radiol.

[68] Barthe N, Chatti K, Coulon P, Maîtrejean S, Basse-Cathalinat B (2004). Nucl Instrum Methods Phys Res, Sect A.

[69] Gahan P (1972). Autoradiography for Biologists.

[70] Santini M A, Ratner C, Aznar S, Klein A B, Knudsen G M, Mikkelsen J D (2013). J Neurosci Res.

[71] Caro L G, van Tubergen R P (1962). J Cell Biol.

[72] Kennel S J, Woodward J D, Rondinone A J, Wall J, Huang Y, Mirzadeh S (2008). Nucl Med Biol.

[73] Kner P, Chhun B B, Griffis E R, Winoto L, Gustafsson M G L (2009). Nat Methods.

[74] Blaise S, Kneib M, Rousseau A, Gambino F, Chenard M-P, Messadeq N, Muckenstrum M, Alpy F, Tomasetto C, Humeau Y (2012). PLoS One.

[75] Cho M, Cho W-S, Choi M, Kim S J, Han B S, Kim S H, Kim H O, Sheen Y Y, Jeong J (2009). Toxicol Lett.

[76] Gopee N V, Roberts D W, Webb P, Cozart C R, Siitonen P H, Warbritton A R, Yu W W, Colvin V L, Walker N J, Howard P C (2007). Toxicol Sci.

[77] Gopee N V, Roberts D W, Webb P, Cozart C R, Siitonen P H, Latendresse J R, Warbitton A R, Yu W W, Colvin V L, Walker N J (2009). Toxicol Sci.

[78] Chu M, Wu Q, Wang J, Hou S, Miao Y, Peng J, Sun Y (2007). Nanotechnology.

[79] Graf C, Gao Q, Schütz I, Noufele C N, Ruan W, Posselt U, Korotianskiy E, Nordmeyer D, Rancan F, Hadam S (2012). Langmuir.

[80] Nabeshi H, Yoshikawa T, Matsuyama K, Nakazato Y, Matsuo K, Arimori A, Isobe M, Tochigi S, Kondoh S, Hirai T (2011). Biomaterials.

[81] Ostrowski A, Nordmeyer D, Boreham A, Brodwolf R, Mundhenk L, Fluhr J W, Lademann J, Graf C, Rühl E, Alexiev U (2014). Nanomedicine.

[82] Boreham A, Kim T-Y, Spahn V, Stein C, Mundhenk L, Gruber A D, Haag R, Welker P, Licha K, Alexiev U (2011). ACS Med Chem Lett.

[83] Choi J, Burns A A, Williams R M, Zhou Z, Flesken-Nikitin A, Zipfel W R, Wiesner U, Nikitin A Y (2007). J Biomed Opt.

[84] Ow H, Larson D R, Srivastava M, Baird B A, Webb W W, Wiesner U (2005). Nano Lett.

[85] Riehemann K, Schneider S W, Luger T A, Godin B, Ferrari M, Fuchs H (2009). Angew Chem, Int Ed.

[86] Medintz I L, Uyeda H T, Goldman E R, Mattoussi H (2005). Nat Mater.

[87] Kim S, Lim Y T, Soltesz E G, De Grand A M, Lee J, Nakayama A, Parker J A, Mihaljevic T, Laurence R G, Dor D M (2004). Nat Biotechnol.

[88] Graf C, Dembski S, Hofmann A, Rühl E (2006). Langmuir.

[89] Gerion D, Pinaud F, Williams S C, Parak W J, Zanchet D, Weiss S, Alivisatos A P (2001). J Phys Chem B.

[90] Dembski S, Graf C, Krüger T, Gbureck U, Ewald A, Bock A, Rühl E (2008). Small.

[91] Haase M, Schäfer H (2011). Angew Chem, Int Ed.

[92] Wang F, Liu X (2009). Chem Soc Rev.

[93] Gorris H H, Wolfbeis O S (2013). Angew Chem, Int Ed.

[94] Choi H S, Ashitate Y, Lee J H, Kim S H, Matsui A, Insin N, Bawendi M G, Semmler-Behnke M, Frangioni J V, Tsuda A (2010). Nat Biotechnol.

[95] Lee C-M, Jeong H-J, Yun K-N, Kim D W, Sohn M-H, Lee J K, Jeong J, Lim S T (2012). Int J Nanomed.

[96] Cheon J, Lee J-H (2008). Acc Chem Res.

[97] Cheng S-H, Li F-C, Souris J S, Yang C-S, Tseng F-G, Lee H-S, Chen C-T, Dong C-Y, Lo L-W (2012). ACS Nano.

[98] van Schooneveld M M, Vucic E, Koole R, Zhou Y, Stocks J, Cormode D P, Tang C Y, Gordon R E, Nicolay K, Meijerink A (2008). Nano Lett.

[99] Bastiaens P I H, Squire A (1999). Trends Cell Biol.

[100] Alexiev U, Rimke I, Pöhlmann T (2003). J Mol Biol.

[101] Alexiev U, Farrens D L (2014). Biochim Biophys Acta.

[102] Festy F, Ameer-Beg S M, Ng T, Suhling K (2007). Mol BioSyst.

[103] Boreham A, Brodwolf R, Pfaff M, Kim T-Y, Schlieter T, Mundhenk L, Gruber A D, Gröger D, Licha K, Haag R (2014). Polym Adv Technol.

[104] Roberts M S, Roberts M J, Robertson T A, Sanchez W, Thörling C, Zou Y, Zhao X, Becker W, Zvyagin A V (2008). J Biophotonics.

[105] Levenson R M, Mansfield J R (2006). Cytometry, Part A.

[106] Prow T W, Grice J E, Lin L L, Faye R, Butler M, Becker W, Wurm E M T, Yoong C, Robertson T A, Soyer H P (2011). Adv Drug Delivery Rev.

[107] Alnasif N, Zoschke C, Fleige E, Brodwolf R, Boreham A, Rühl E, Eckl K-M, Merk H-F, Hennies H C, Alexiev U (2014). J Controlled Release.

[108] Zimmermann T, Rietdorf J (2005). Spectral Imaging and Linear Unmixing in Light Microscopy. Microscopy Techniques.

[109] Gao X, Cui Y, Levenson R M, Chung L W K, Nie S (2004). Nat Biotechnol.

[110] Zimmermann T, Rietdorf J, Pepperkok R (2003). FEBS Lett.

[111] Mansfield J R, Hoyt C, Levenson R M (2001). Visualization of Microscopy-Based Spectral Imaging Data from Multi-Label Tissue Sections. Current Protocols in Molecular Biology.

[112] Mansfield J R (2014). Med Lab Obs.

[113] Mortensen L J, Oberdörster G, Pentland A P, DeLouise L A (2008). Nano Lett.

[114] Levenson R M, Lynch D T, Kobayashi H, Backer J M, Backer M V (2008). ILAR J.

[115] Gustafsson M G L (2000). J Microsc (Oxford, U K).

[116] Gustafsson M G L (2005). Proc Natl Acad Sci U S A.

[117] Habuchi S (2014). Front Bioeng Biotechnol.

[118] Heintzmann R, Gustafsson M G L (2009). Nat Photonics.

[119] York A G, Chandris P, Nogare D D, Head J, Wawrzusin P, Fischer R S, Chitnis A, Shroff H (2013). Nat Methods.

[120] Gustafsson M G L, Shao L, Carlton P M, Wang C J R, Golubovskaya I N, Cande W Z, Agard D A, Sedat J W (2008). Biophys J.

[121] Schermelleh L, Carlton P M, Haase S, Shao L, Winoto L, Kner P, Burke B, Cardoso M C, Agard D A, Gustafsson M G L (2008). Science.

[122] Leung B O, Chou K C (2011). Appl Spectrosc.

[123] Min J, Jang J, Keum D, Ryu S-W, Choi C, Jeong K-H, Ye J C (2013). Sci Rep.

[124] Fernández-Suárez M, Ting A Y (2008). Nat Rev Mol Cell Biol.

[125] Lidke K, Rieger B, Jovin T, Heintzmann R (2005). Opt Express.

[126] Chernenko T, Mätthaus C, Milane L, Quintero L, Amiji M, Diem M (2009). ACS Nano.

[127] Garrett N L, Lalatsa A, Uchegbu I, Schätzlein A, Moger J (2012). J Biophotonics.

[128] Rodriguez L G, Lockett S J, Holtom G R (2006). Cytometry, Part A.

[129] Chernenko T, Buyukozturk F, Miljkovic M, Carrier R, Diem M, Amiji M (2013). Drug Delivery Transl Res.

[130] Wartewig S, Neubert R H H (2005). Adv Drug Delivery Rev.

[131] Nijssen A, Koljenović S, Schut T C B, Caspers P J, Puppels G J (2009). J Biophotonics.

[132] Krafft C, Dietzek B, Popp J (2009). Analyst.

[133] Cialla D, März A, Böhme R, Theil F, Weber K, Schmitt M, Popp J (2012). Anal Bioanal Chem.

[134] Belsey N A, Garrett N L, Contreras-Rojas L R, Pickup-Gerlaugh A J, Price G J, Moger J, Guy R H (2014). J Controlled Release.

[135] Freudiger C W, Min W, Saar B G, Lu S, Holtom G R, He C, Tsai J C, Kang J X, Xie X S (2008). Science.

[136] König K, Breunig H G, Bückle R, Kellner-Höfer M, Weinigel M, Büttner E, Sterry W, Lademann J (2011). Laser Phys Lett.

[137] Schlücker S (2009). ChemPhysChem.

[138] Moger J, Johnston B D, Tyler C R (2008). Opt Express.

[139] Hanlon E B, Manoharan R, Koo T W, Shafer K E, Motz J T, Fitzmaurice M, Kramer J R, Itzkan I, Dasari R R, Feld M S (2000). Phys Med Biol.

[140] Li M, Xu J, Romero-Gonzalez M, Banwart S A, Huang W E (2012). Curr Opin Biotechnol.

[141] Krafft C, Dietzek B, Schmitt M, Popp J (2012). J Biomed Opt.

[142] Leung B O, Brash J L, Hitchcock A P (2010). Materials.

[143] Andrews J C, Meirer F, Liu Y, Mester Z, Pianetta P (2011). Microsc Res Tech.

[144] Gilbert B, Fakra S C, Xia T, Pokhrel S, Mädler L, Nel A E (2012). ACS Nano.

[145] Chien C-C, Cheng C-C, Chen H H, Hwu Y, Chu Y S, Petibois C, Chen A, Ching Y-T, Margaritondo G (2012). Anal Bioanal Chem.

[146] Schneider G, Guttmann P, Heim S, Rehbein S, Mueller F, Nagashima K, Heymann J B, Müller W G, McNally J G (2010). Nat Methods.

[147] Hitchcock A P, Dynes J J, Johansson G, Wang J, Botton G (2008). Micron.

[148] Ade H, Hitchcock A P (2008). Polymer.

[149] Eichert D, Gregoratti L, Kaulich B, Marcello A, Melpignano P, Quaroni L, Kiskinova M (2007). Anal Bioanal Chem.

[150] Sedlmair J, Gleber S-C, Mert S Ö, Bertilson M, von Hofsten O, Thieme J, Pfohl T (2011). Microsc Microanal.

[151] Graf C, Meinke M, Gao Q, Hadam S, Raabe J, Sterry W, Blume-Peytavi U, Lademann J, Rühl E, Vogt A (2009). J Biomed Opt.

[152] Fagerland J A, Wall H G, Pandher K, LeRoy B E, Gagne G D (2012). Toxicol Pathol.

[153] Gontier E, Ynsa M-D, Bíró T, Hunyadi J, Kiss B, Gáspár K, Pinheiro T, Silva J-N, Filipe P, Stachura J (2008). Nanotoxicology.

[154] Kempen P J, Thakor A S, Zavaleta C, Gambhir S S, Sinclair R (2013). Microsc Microanal.

[155] Mercer R R, Hubbs A F, Scabilloni J F, Wang L, Battelli L A, Friend S, Castranova V, Porter D W (2011). Part Fibre Toxicol.

[156] Samberg M E, Oldenburg S J, Monteiro-Riviere N A (2010). Environ Health Perspect.

[157] Yamashita K, Yoshioka Y, Higashisaka K, Mimura K, Morishita Y, Nozaki M, Yoshida T, Ogura T, Nabeshi H, Nagano K (2011). Nat Nanotechnol.

[158] Petri-Fink A, Steitz B, Finka A, Salaklang J, Hofmann H (2008). Eur J Pharm Biopharm.

[159] Haase A, Rott S, Mantion A, Graf P, Plendl J, Thünemann A F, Meier W P, Taubert A, Luch A, Reiser G (2012). Toxicol Sci.

[160] Droste M S, Biel S S, Terstegen L, Wittern K-P, Wenck H, Wepf R (2005). J Biomed Opt.

[161] Richter T, Biel S S, Sattler M, Wenck H, Wittern K P, Wiesendanger R, Wepf R J (2007). J Microsc (Oxford, U K).

[162] Echlin P (1992). Low-temperature microscopy and analysis.

[163] Lucas M S, Günthert M, Gasser P, Lucas F, Wepf R, Thomas M-R, Paul V (2012). Bridging Microscopes: 3D Correlative Light and Scanning Electron Microscopy of Complex Biological Structures. Correlative Light and Electron MIcroscopy.

[164] Norlén L (2007). Int J Cosmet Sci.

[165] Kuntsche J, Horst J C, Bunjes H (2011). Int J Pharm.

[166] Kunisawa J, Masuda T, Katayama K, Yoshikawa T, Tsutsumi Y, Akashi M, Mayumi T, Nakagawa S (2005). J Controlled Release.

[167] Iannuccelli V, Coppi G, Romagnoli M, Sergi S, Leo E (2013). Int J Pharm.

[168] Egerton R F (2009). Rep Prog Phys.

[169] Jeong S H, Kim J H, Yi S M, Lee J P, Kim J H, Sohn K H, Park K L, Kim M-K, Son S W (2010). Biochem Biophys Res Commun.

[170] George R, Merten S, Wang T T, Kennedy P, Maitz P (2014). Australas J Dermatol.

[171] Asharani P V, Wu Y L, Gong Z, Valiyaveettil S (2008). Nanotechnology.

[172] Patri A, Umbreit T, Zheng J, Nagashima K, Goering P, Francke-Carroll S, Gordon E, Weaver J, Miller T, Sadrieh N (2009). J Appl Toxicol.

[173] Sousa A A, Leapman R D (2012). Ultramicroscopy.

[174] Pawley J (1997). Scanning.

[175] Walczak A P, Fokkink R, Peters R, Tromp P, Herrera Rivera Z E, Rietjens I M C M, Hendriksen P J M, Bouwmeester H (2013). Nanotoxicology.

[176] Købler C, Saber A T, Jacobsen N R, Wallin H, Vogel U, Qvortrup K, Mølhave K (2014). Anal Bioanal Chem.

[177] Muscariello L, Rosso F, Marino G, Giordano A, Barbarisi M, Cafiero G, Barbarisi A (2005). J Cell Physiol.

[178] Chang H-H, Cheng C-L, Huang P-J, Lin S-Y (2014). Anal Bioanal Chem.

[179] (2014). SU8000 Series UHR Cold-Emission FE-SEM | Hitachi High Technologies America, Inc..

[180] Mercer R R, Hubbs A F, Scabilloni J F, Wang L, Battelli L A, Schwegler-Berry D, Castranova V, Porter D W (2010). Part Fibre Toxicol.

[181] Richter T, Peuckert C, Sattler M, Koenig K, Riemann I, Hintze U, Wittern K-P, Wiesendanger R, Wepf R (2004). Skin Pharmacol Physiol.

[182] Sung B, Kim M S, Lee B-C, Yoo J S, Lee S-H, Kim Y-J, Kim K-W, Soh K-S (2008). Naturwissenschaften.

[183] Win K Y, Feng S-S (2005). Biomaterials.

[184] Mahe B, Vogt A, Liard C, Duffy D, Abadie V, Bonduelle O, Boissonnas A, Sterry W, Verrier B, Blume-Peytavi U (2008). J Invest Dermatol.

[185] Labouta H I, Kraus T, El-Khordagui L K, Schneider M (2011). Int J Pharm.

